# GABA, glutamine, glutamate oxidation and succinic semialdehyde dehydrogenase expression in human gliomas

**DOI:** 10.1186/s13046-018-0946-5

**Published:** 2018-11-07

**Authors:** Zoltán Hujber, Gergő Horváth, Gábor Petővári, Ildikó Krencz, Titanilla Dankó, Katalin Mészáros, Hajnalka Rajnai, Norbert Szoboszlai, William P. J. Leenders, András Jeney, László Tretter, Anna Sebestyén

**Affiliations:** 1 1st Department of Pathology and Experimental Cancer Research, Semmelweis University, Üllői út 26, Budapest, 1085 Hungary; 2 Department of Medical Biochemistry, MTA-SE Laboratory for Neurobiochemistry, Semmelweis University, Budapest, 1444 Hungary; 3 Hungarian Academy of Sciences - Momentum Hereditary Endocrine Tumours Research Group, Semmelweis University - National Bionics Program, Budapest, 1088 Hungary; 4 Laboratory of Environmental Chemistry and Bioanalytics, Department of Analytical Chemistry, Institute of Chemistry, Eötvös Loránd University, Budapest, 1518 Hungary; 5 Department of Biochemistry, Radboud University Medical Center, Nijmegen, The Netherlands

**Keywords:** Glioma, Bioenergetics, IDH1 mutation, 2-hydroxyglutarate, Glutamine, GABA, Succinic semialdehyde dehydrogenase

## Abstract

**Background:**

Bioenergetic characterisation of malignant tissues revealed that different tumour cells can catabolise multiple substrates as salvage pathways, in response to metabolic stress. Altered metabolism in gliomas has received a lot of attention, especially in relation to IDH mutations, and the associated oncometabolite D-2-hydroxyglutarate (2-HG) that impact on metabolism, epigenetics and redox status. Astrocytomas and oligodendrogliomas, collectively called diffuse gliomas, are derived from astrocytes and oligodendrocytes that are in metabolic symbiosis with neurons; astrocytes can catabolise neuron-derived glutamate and gamma-aminobutyric acid (GABA) for supporting and regulating neuronal functions.

**Methods:**

Metabolic characteristics of human glioma cell models – including mitochondrial function, glycolytic pathway and energy substrate oxidation – in relation to IDH mutation status and after 2-HG incubation were studied to understand the Janus-faced role of IDH1 mutations in the progression of gliomas/astrocytomas. The metabolic and bioenergetic features were identified in glioma cells using wild-type and genetically engineered IDH1-mutant glioblastoma cell lines by metabolic analyses with Seahorse, protein expression studies and liquid chromatography-mass spectrometry.

**Results:**

U251 glioma cells were characterised by high levels of glutamine, glutamate and GABA oxidation. Succinic semialdehyde dehydrogenase (SSADH) expression was correlated to GABA oxidation. GABA addition to glioma cells increased proliferation rates. Expression of mutated IDH1 and treatment with 2-HG reduced glutamine and GABA oxidation, diminished the pro-proliferative effect of GABA in SSADH expressing cells. SSADH protein overexpression was found in almost all studied human cases with no significant association between SSADH expression and clinicopathological parameters (e.g. IDH mutation).

**Conclusions:**

Our findings demonstrate that SSADH expression may participate in the oxidation and/or consumption of GABA in gliomas, furthermore, GABA oxidation capacity may contribute to proliferation and worse prognosis of gliomas. Moreover, IDH mutation and 2-HG production inhibit GABA oxidation in glioma cells. Based on these data, GABA oxidation and SSADH activity could be additional therapeutic targets in gliomas/glioblastomas.

**Electronic supplementary material:**

The online version of this article (10.1186/s13046-018-0946-5) contains supplementary material, which is available to authorized users.

## Introduction

Gliomas, glial cell derived central nervous system malignancies, are a heterogeneous, aggressive tumour type with poor prognosis. The incidence of isocitrate dehydrogenase (IDH) mutations is high in low-grade gliomas. Despite the fact that these malignancies are still incurable, patients with IDH-mutant gliomas have a better prognosis and response to chemo-and radiotherapy than patients with IDH wild-type tumours [1, 2]. IDH mutations can also be implicated in the formation of other tumour types (acute myeloid leukaemia – AML, chondrosarcomas, intrahepatic cholangiocarcinoma – ICC). In these non-glial malignancies, IDH mutations appear to confer a worse prognosis to the patient; although there is some controversy in case of ICC and AMLs [3, 4].

Based on highly detailed analyses of the genetic basis for malignant progression (gene amplifications, mutations, loss of chromosome arms, gene expression, DNA methylation status), several altered pathways in IDH-mutated gliomas were characterised, including RTK-PI3K-mTOR, Notch signalling, cell cycle and DNA damage response regulation. These studies concluded that IDH mutation is at the centre of epigenetic alterations in glioma cells [5, 6]. The prevalence of IDH mutation is high in grade II-III and secondary glioblastomas (70–80%), the most frequently (> 90%) mutated IDH isoform is the cytosolic IDH1 R132H [7, 8], that has gained a neomorphic activity resulting in conversion of α-ketoglutarate (aKG) to the oncometabolite D-2-hydroxyglutarate (2-HG). In addition, the mutation has a negative impact on the function on the wild-type allele [9].

2-HG can accumulate to millimolar concentrations in cells and in the extracellular environment [10, 11]. Moreover, mutated IDH1 not only causes 2-HG production, but also initiates changes in metabolic adaptation of mutated cells [12]. Mutant IDH1 oxidises NADPH to NADP^+^ during 2-HG production, instead of reducing NADP^+^ to NADPH as is done by the wild-type IDH1 enzyme during conversion of isocitrate to aKG. Because 2-HG and aKG are structurally very similar metabolites, 2-HG acts as a competitive inhibitor of aKG-dependent dioxygenases including prolyl-hydroxylases, histone- and DNA demethylases [13–15]. Cancer-related metabolic alterations, adaptations and their role in tumourigenesis and progression are extensively studied in many cancers. The metabolic characterisation of human gliomas started with the reinterpretation of Warburg effect suggesting that other substrates than glucose can also be oxidised by glioma cells [12, 16, 17], such as glutamine and acetate [18–20].

Multiple substrates can fill up the TCA cycle – which is in the centre of metabolic activity – to provide acetyl-CoA; and many studies reported that cells can use glutamine and/or glutamate as catabolic substrates [21–23]. Previous results suggested that glioma cells adaptively run the TCA cycle backwards to power the electron transport chain, especially in case of IDH-mutant cells. Analogous to fumarate hydratase (FH) and succinate dehydrogenase (SDH) mutations, which can also cause oncometabolite production, IDH mutation provides an example of how a single mutation can reprogram cellular metabolism and affect pathobiology [13, 15, 24].

Recent years have yielded exciting findings in the field of tumour metabolism, and some aspects of these metabolic alterations related to IDH mutations have also been described [25, 26].

Astrocytes and GABA-ergic and glutamatergic neurons play roles in the maintenance of the neurotransmitter pools of glutamate and gamma-aminobutyric acid (GABA). Astrocytes and neurons are in metabolic symbiosis, astrocytes can catabolise glutamate and GABA for supporting and regulating neuronal functions. GABA can feed the TCA cycle via the activities of GABA transaminase and succinic semialdehyde dehydrogenase (SSADH) to produce succinate [27]. The expression changes of GABA utilisation enzymes and GABA consumption were only studied in few recent publications in cancer research [28, 29].

An emerging question is whether glioma cells have the ability to use the GABA shunt to support energy production and proliferation. This indicates a need to understand the effect of produced 2-HG (caused by IDH1 mutations) on the former pathway.

Our study provides an exciting opportunity to advance our knowledge of the effects of IDH1 mutation and 2-HG accumulation to identify several mechanisms which can help to understand the Janus-faced role of IDH1 mutations in the progression of gliomas – IDH mutations could be driver oncogenic alterations, however, IDH-mutant human gliomas have better prognosis than wild-type. In the present study, the alterations of bioenergetic characteristics and different substrate oxidation capacities in relation to the expressions of relevant proteins in glioma cell lines were analysed (Additional file 1: Figure S1 summarises the analysed pathways). Our presented results demonstrate that SSADH expression – an IDH mutation independent in vivo characteristic of human glioma cells – provides a possibility for GABA oxidation. This may have special importance in survival, proliferation and metabolic adaptation of glioma cells.

## Methods

All materials were purchased from Merck-Sigma-Aldrich (Darmstadt, Germany), except where it is indicated in the text.

### In vitro cell cultures

U251 MG (U251 wt), genetically engineered U251 MG mutant IDH1 R132H overexpressing (U251 IDH1m) [25], U87 MG (ATCC) and U373 MG (kindly provided by G. Sáfrány) human astrocytoma/glioblastoma cells were cultured in DMEM high glucose medium supplemented with 10% foetal bovine serum, 2 mM L-glutamine and 100 UI/ml penicillin-streptomycin. R132H mutation status was confirmed by immunocytochemistry using anti-IDH1 R132H (Dianova, H09 clone, 1:80) antibody in different cell lines after paraformaldehyde (4%) fixation. For metabolic flux experiments, cells were washed with DMEM D5030 (glucose-, glutamine- and pyruvate-free medium) followed by incubation with DMEM D5030 medium containing U-^13^C-glucose, U-^13^C-glutamine or 2-^13^C-acetate (Cambridge Isotope Laboratories Inc.). T25 or T75 flasks (for Western blot, liquid chromatography-mass spectrometry (LC-MS) and ^13^C-labelling experiments) and 96-well plates (sulforhodamine-B and Alamar Blue proliferation assays), special Seahorse plates were used with appropriate cell numbers (3–8 × 10^5^ cells/flask or 2.5 × 10^3^ – 5 × 10^4^ cells/well) for different treatments and measurements. To analyse the direct effect of 2-HG accumulation and the role of GABA metabolism in U251 wt cells, 4 mM 2-HG, 5 mM GABA and 0.6 mM vigabatrin were added for various time periods (24 h, 72 h and 14/28 days). We selected concentrations based on previous publications.

### Cell proliferation measurements

Beside cell counting the proliferation rate was analysed by sulforhodamine-B (SRB) assay which correlates to the protein content of the analysed samples. For 72-h proliferation assays 2.5 × 10^3^ untreated or pre-treated glioma cells/100 μl well were seeded; followed by incubation under different conditions. Thereafter the cells were fixed with 10% trichloroacetic acid subsequently stained with SRB using 10 mM Tris, and the absorbance was measured at 570 nm. In addition, Alamar Blue (Thermo Fisher Scientific) test was also used for proliferation studies. The incubation period was 4 h, fluorescence was measured by Fluoroskan Ascent FL fluorimeter software (Labsystems International). Percentages of proliferation were given relative to control samples.

### Expression analysis of different proteins by Western blot

Protein concentrations of cell extracts were measured with using Bradford assay (Biorad). Equal amounts of proteins were separated by 8–12% SDS-PAGE gels following their transfer to PVDF membrane applying BioRad Trans Blot Cell. Membranes were stained with Ponceau S, before incubation the following primary antibodies were used in expression studies: anti-phosphofructokinase P (PFKP) (Cell Signaling 1:1000 #8164); anti-hexokinase 2 (HK2) (Cell Signaling 1:1000 #2867); anti-β-F1-ATPase (ATPB) (Abcam 1:2000 #14370), anti-glutaminase (Gls) (Abcam 1:1000 #156876); anti-acetyl-CoA synthetase 2 (ACSS2) (Cell Signaling 1:1000 #3658); anti-alanine, serine, cysteine-preferring transporter 2 (ASCT2) (Bethyl 1:2000 #A304-353A); anti-succinic semialdehyde dehydrogenase (SSADH) (Abcam 1:1000 #129017); anti-GABA transporter 1 (GAT1) (Abcam 1:500 #426). Finally, biotinylated secondary antibodies, Vectastain Elite ABC Kit (Vector) and enhanced chemiluminescence technique (Thermo Fisher Scientific ECL Western Blotting Substrate) were applied by using C-Digit System (Li Cor Biosciences) detection equipment. The membranes were stripped and probed by anti-β-actin antibody (Sigma-Aldrich; #A228; 1:5000), to prove equal protein loading as well.

### Assay to detect cellular respiration and extracellular pH changes

Real-time measurements of oxygen consumption rate (OCR), reflecting mitochondrial oxidation and extracellular acidification rate (ECAR), indicated as a parameter of glycolytic activity, were performed on a Seahorse XF96 Analyzer (Agilent Technologies, USA) based on previous descriptions [30–32].

Glioma cell lines were plated in 100 μl complete DMEM growth media at 1.5 × 10^4^ cells/well density onto 96-well Seahorse plates (Agilent Technologies, USA) 24 h prior to the assays. The medium was removed and was replaced by glucose-, glutamine- and pyruvate-free DMEM medium (D5030 pH 7.4). The basal OCR and ECAR were calculated via XF96 Analyzer software (Agilent Technologies, USA) after 1.5-h incubation at this condition.

Following this, substrate utilisation was measured by using different substrates in parallel wells.

During the measurements freshly prepared substrates (glucose 10 mM, glutamine 2.5 mM, citrate 5 mM, GABA 5 mM, lactate 5 mM, malate 10 mM, acetate 10 mM and glutamate 5 mM) and/or metabolic inhibitors/modulators (oligomycin 2 μM, 2,4-dinitrophenol - DNP 100 μM and antimycin A + rotenone 1–1 μM) were injected into each well to reach the desired final working concentration.

### LC-MS/MS method for quantitative metabolite analysis

Intra- or extracellular metabolites - citrate, aKG, succinate, fumarate, malate, glutamate, 2-HG - were extracted by a modified method based on Szoboszlai et al. [33, 34]. Cells (minimum 5 × 10^5^ cells) were quenched in liquid nitrogen. Metabolites were extracted from cells and in parallel 300–500 μl supernatant by methanol-chloroform-H_2_O (9:1:1) and vortexed at 4 °C. After centrifugation (15,000×g, 10 min, 4 °C) the clear supernatants were stored at -80 °C until measurements. Citrate, aKG, succinate, fumarate, malate, glutamate and 2-HG concentrations were assessed by using calibration curves obtained with the dilution of analytical purity standards in the range of 0.5–50 μM. LC-MS grade water, LC-MS grade methanol and LC-MS grade formic acid were purchased from VWR International Ltd. (Debrecen, Hungary).

LC-MS/MS assays were performed on a Perkin-Elmer Flexar FX10 ultra-performance liquid chromatograph coupled with a Sciex 5500 QTRAP mass spectrometer. For chromatographic separation a Phenomenex Luna Omega C18 column, (100 × 2.1 mm, 1.6 μm) was used (GenLab Ltd., Budapest, Hungary). The mobile phase consisted of water containing 0.1% (*v*/v) formic acid (A) and methanol containing 0.1% (v/v) formic acid (B). The mass spectrometer was operating in negative electrospray ionisation mode with the following settings: source temperature: 300 °C ionisation voltage: − 4000 V, curtain gas: 35 psi, gas1: 35 psi, gas2: 35 psi, entrance potential: -10 V, CAD gas: medium. Quantitative analysis was performed in multiple reaction monitoring (MRM) mode.

### Different substrate consumptions were analysed using ^13^C-labelling and LC-MS measurements

Sub-confluent cells were washed and incubated in DMEM D5030 medium with 10 mM D-U-^13^C-glucose or 4 mM L-U-^13^C-glutamine or 10 mM 2-^13^C-acetate (Cambridge Isotope Laboratories, Andover, MA, USA) addition for 1 h in short-time labelling experiments before the extraction [32]. In 24-h labelling experiments – avoiding long-term starvation/non-physiological conditions – cells were seeded in DMEM D5030 medium supplemented with FBS, and the combination of labelled and/or unlabelled glucose (10 mM) and glutamine (4 mM) or acetate (10 mM) were added to cell cultures for 24 h.

### SSADH expression analysis by immunohistochemistry (tissue microarray) in clinical samples

47 glioma patients (type: astrocytoma *n* = 14, oligodendroglioma *n* = 14, glioblastoma *n* = 19; sex: female *n* = 23, male = 24; WHO grade: II *n* = 9, III *n* = 19, IV *n* = 19; IDH1 R132H mutation: positive *n* = 32, negative *n* = 15) were included in a tissue microarray (TMA) study. Three normal brain and renal tissues were included as controls. Expression studies by IHC on biopsy materials were approved by the Institutional Ethical Review Board (TUKEB no. 7/2006). TMA sections were deparaffinised, endogenous peroxidase blocking was followed by antigen retrieval (sodium citrate; pH 6). Immunostaining was performed by routine diagnostic antibodies (such as anti-IDH1 R132H), SSADH (Abcam #129017 1:500) antibody and Novolink Polymer Detection System (Novocastra, Wetzlar, Germany), visualised by DAB and counterstained with haematoxylin. H-scores (scale of 0–300) were determined by two independent pathologists with semi quantitative analysis of immunoreactivity using 3DHistech Pannoramic Viewer program according to Krencz et al. [35]. The H-score was calculated from staining intensity (0, 1+, 2+, or 3+) and positively stained tumour cells (percentage). The final H-score was calculated by averaging the H-scores of all the scores from the same tissue. SSADH expression was categorised as low (H-score 0–149) and high (H-score 150–300).

### Statistical analysis

The data are presented as mean with standard deviation and calculated from three independent experiments with minimum three or more parallels depending on the method used. Results were statistically evaluated through one-way ANOVA with post-hoc Tukey’s and Dunnett’s tests for multiple comparisons by IBM SPSS Statistics software, version 22 (SPSS Inc.). Mann-Whitney U test and Kruskal-Wallis test were used to compare SSADH expressions and clinicopathologic parameters in human gliomas using IBM SPSS Statistics software, version 22 (SPSS Inc.). *p* < 0.05 was considered statistically significant.

## Results

### Characteristics of cellular respiration and glycolytic activity in glioma cells

**Fig. 1:**
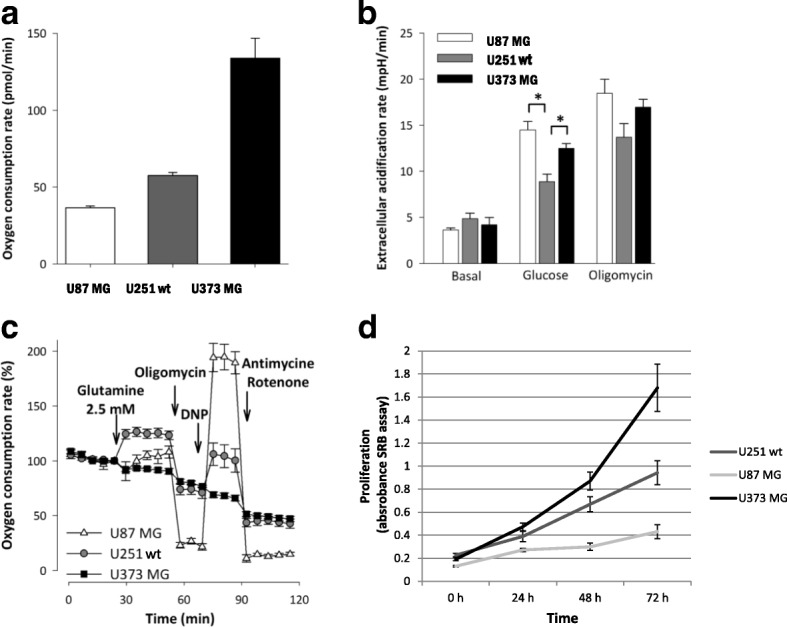
Oxygen consumption, extracellular acidification rate and glutamine oxidation differences were measured by Seahorse in U87 MG, U251 wt, U373 MG glioma cell lines using D5030 medium. **a** Different oxygen consumption rates in the studied cell lines (pmol/min). **b** Extracellular acidification rates were measured in minimal medium (D5030) at basal rate and after glucose (10 mM) addition following oligomycin (2 μM) treatment. **c** Oxygen consumption rate curves in minimal medium (D5030), and after glutamine (2.5 mM) addition and using several metabolic inhibitors and modulators (oligomycin - 2 μM; 2,4-dinitrophenol - DNP - 100 μM; antimycin - 1 μM - and rotenone - 1 μM) *:*p* < 0.05. **d** Time-dependent growth of different glioma cell lines (U87 MG, U251 wt and U373 MG) at normal non-starving conditions. Cell proliferation was monitored by SRB test using 2.5 × 10^3^ cells/100 μl at the starting point of the experiments

The basal oxygen consumption rates (OCR) among the investigated gliomas showed significant differences. The lowest respiration rate was observed in U87 MG, while the highest basal rate was produced by U373 MG cells in DMEM D5030 medium (Fig. 1a). In contrast with the respiratory parameters, there were no differences in the basal extracellular acidification (ECAR) rates. However, glucose addition reveals alteration in the metabolic adaptation of gliomas. Lactate productions using glucose administration with or without mitochondrial respiratory inhibitor were the highest in U87 MG cells, which have the lowest basal respiratory activity (Fig. 1b). These findings indicate an inverse relationship between the basal oxygen consumption and the glycolytic activity. Additionally, we compared the glutamine metabolising properties of the examined gliomas. The glutaminolysis-driven respiratory capacity was significantly higher in U251 wt cells than in U87 MG or U373 MG cells (Fig. 1c). Using same cell density, time-dependent growth showed individual differences in these glioma cells (Fig. 1d), but these did not correlate to their oxygen consumptions. U373 MG cells have the fastest proliferation and these cells have both high glucose utilisation and high respiratory capacity.

### IDH1 mutation and related 2-HG accumulation increase the basal respiration and decrease the glycolytic capacity

**Fig. 2:**
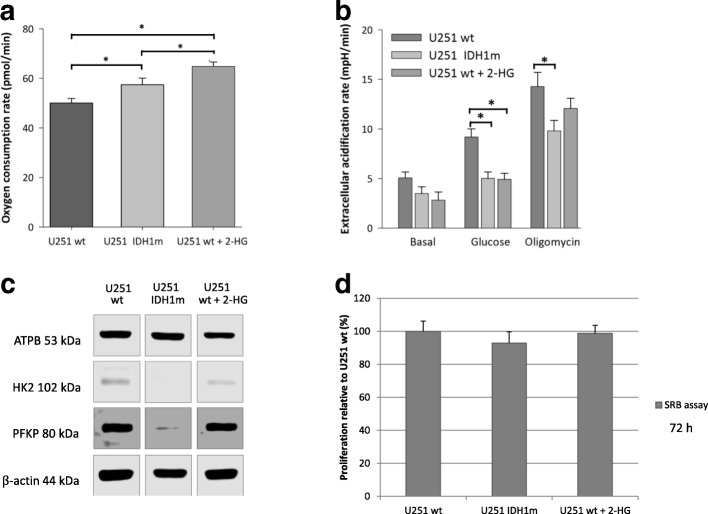
IDH1 mutation (IDH1m) and the elevated 2-HG level related metabolic alterations in U251 glioma cells (oxygen consumption and metabolic enzyme expression differences). **a** Oxygen consumption rates in U251 wt, U251 IDH1m and 2-HG pre-treated (72 h, 4 mM) U251 wt cells (OCR was measured in D5030 medium by Seahorse). **b** Extracellular acidification data in minimal medium (D5030), and after glucose (10 mM) addition and using oligomycin (2 μM) thereafter (Seahorse measurements). **c** Alterations in the expression of different metabolic enzymes in IDH1m and 2-HG-treated (72 h, 4 mM) U251 glioma cells (representative Western blot results using anti-β-F1-ATPase - ATPB, anti-hexokinase 2 - HK2, anti-phosphofructokinase P - PFKP and β-actin). **d** Differences in proliferation of U251 wt, IDH1m and 2-HG pre-treated (72 h) wt cells. The relative proliferation was calculated related to U251 wt (100%), the growing capacity was monitored by SRB tests using 2.5 × 10^3^ cells/100 μl at the starting point of the experiments; *:*p* < 0.05

The effects of the most frequent IDH1 mutation (R132H) of gliomas in energy metabolism and substrate consumption were studied in an IDH1 mutation (R132H) carrying glioma model (isogenic pair: U251 wt and its genetically engineered counterpart - U251 IDH1m). Besides, we studied the short-term effects of 2-HG incubation in U251 wt cells. Comparing the basal respiration of wt, 2-HG-treated wt and IDH1m U251 cells, significantly elevated OCR was observed in case of IDH1m and 2-HG-treated wt cells (Fig. 2a). In contrast, higher extracellular acidification rates – especially after glucose addition – were measured in U251 wt cells compared to IDH1m and 2-HG-treated U251 wt cells (Fig. 2b). These alterations were detected by Seahorse measurements, the altered characteristics related to IDH1 mutation and 2-HG treatment were supported by Western blot analyses of certain metabolic enzyme expressions in these models (Fig. 2c). Higher β-F1-ATPase expression was detected in IDH1m cells in parallel with increased OCR compared to wt cells. However, short-term 2-HG addition did not elevate the expression level of β-F1-ATPase in U251 wt cells. Analysing certain key enzymes of glycolysis (e.g. hexokinase 2, phosphofructokinase P), decrease in their expression was detected in IDH1m and 2-HG-treated wt cells.

U251 wt and IDH1m cells have significantly different in vitro growth characteristics. The proliferation of mutant cells was lower than of wild-type cells registered by cell number during culturing and SRB assay (doubling times were calculated based on Roth V. et al. – [36] – the doubling time of IDH1m cells was 1 h longer than wt by both cell counting and proliferation assay. However, 72-h (short-term) 2-HG treatment had no significant growing effect on cell proliferation rates in U251 wt cultures (Fig. 2d).

### Intracellular metabolite concentration differences and the main source of 2-HG production

**Fig. 3:**
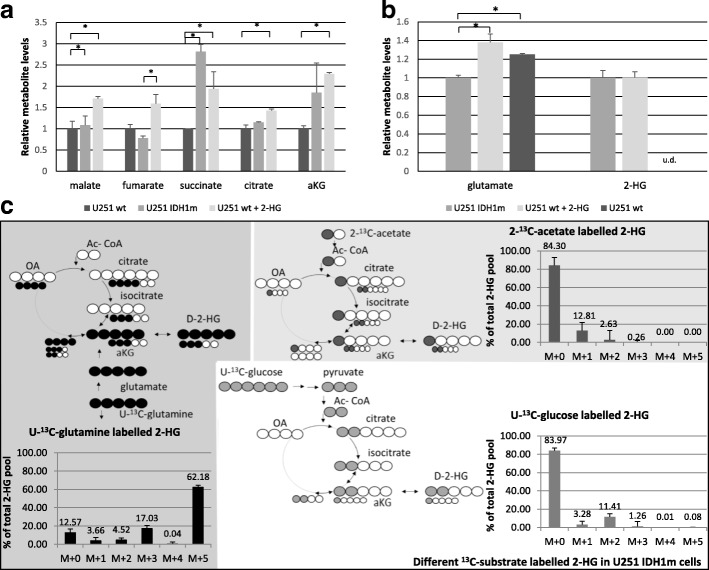
Altered metabolite concentrations in IDH1-mutant and 2-HG-treated (72 h, 4 mM) U251 wt cells and the evaluation of the bioenergetic source of 2-HG production in U251 IDH1m cells (LC-MS measurements). **a** TCA cycle related metabolite levels (relative to wt) in IDH1m and 2-HG-treated U251 wt cells - (DMEM high glucose medium + 10% FBS + 2 mM L- glutamine). **b** Alterations in intracellular glutamate and 2-HG levels (relative to IDH1m) in 2-HG-treated U251 wt cells and in wt cells; u.d. = under detectable level - (DMEM high glucose medium 10% FBS + 2 mM L- glutamine). **c** Unlabelled and ^13^C-labelled intracellular 2-HG productions (% of total 2-HG pool) (24-h labelling period). To avoid starving condition, the medium was supplemented with 10% FBS and 10 mM D-glucose and/or 4 mM L-glutamine: 4 mM U-^13^C-glutamine labelling in D5030 + 10% FBS and 10 mM D-glucose; 10 mM U-^13^C-glucose labelling in D5030 + 10% FBS and 4 mM L-glutamine; or 10 mM 2-^13^C-acetate labelling in D5030 medium + 10% FBS + 10 mM D-glucose and 4 mM L-glutamine. Unlabelled 2-HG does not contain incorporated ^13^C atoms, M + 1/2/3/4/5 = mass number increased with 1/2/3/4 or 5 ^13^C atoms in 2-HG from different labellings; *:*p* < 0.05

The intracellular metabolite concentrations (measured by LC-MS) confirmed the metabolic differences detected among U251 cell culture systems (Fig. 3a). High level of 2-HG was measured in IDH1m cells and similar 2-HG level was observed in 2-HG-treated wt cells by LC-MS (Fig. 3b). Moreover, elevated levels of TCA metabolites were observed in IDH1m and 2-HG-treated U251 wt cells (Fig 3a). Inverse alterations were detected in glutamate concentrations. The amount of glutamate was decreased in IDH1m, but it was increased in 2-HG-treated wt cells comparing to wt cells (Fig. 3b).

To identify which substrates are the prominent sources for 2-HG production in IDH1-mutant U251 cells, the cells were fed with ^13^C-labelled energy substrates (U-^13^C-glucose, U-^13^C-glutamine, 2-^13^C-acetate) in D5030 medium. Based on our 1-h-labelling results, both glutamine and glucose could be sources of 2-HG (5.5% ^13^C-labelled 2-HG from glutamine and ~ 2% from glucose were detected). Performing 24-h-labelling experiments, glutamine was proven to be the main source of 2-HG – 87.43% (sum-total = M + n isotopomers) of the total intracellular 2-HG pool containing glutamine-derived ^13^C-labelling (Fig. 3c), as compared to ^13^C-incorporation percentages of 15.95 and 15.7% from glucose and acetate, respectively (Fig. 3c). Labelling intensities of extracellular 2-HG levels correlated to and followed the intracellular ones in our measurements (Additional file 2: Figure S2).

### IDH1 mutation and 2-HG related different substrate - including GABA - oxidation in U251 MG models

**Fig. 4:**
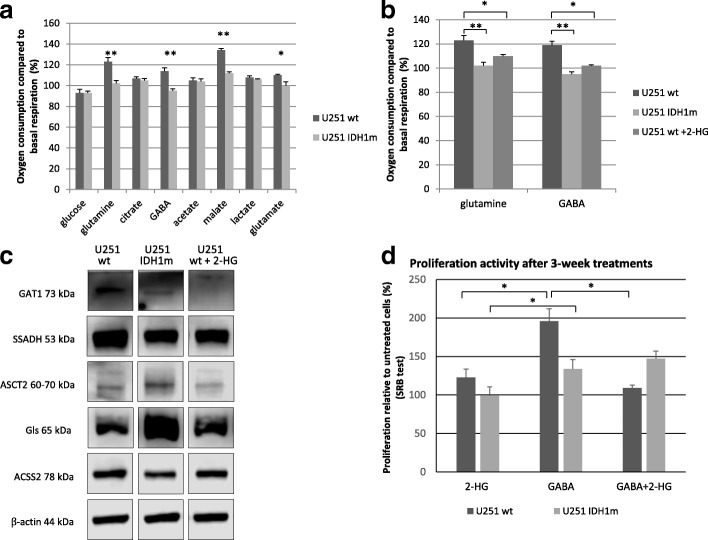
Energy substrate oxidation differences in wt, IDH1m and 2-HG-treated U251 wt cells. **a** Oxygen consumption compared to basal respiration (%) after using different energy substrates: glucose (10 mM), glutamine (2.5 mM), citrate (5 mM), GABA (5 mM), acetate (10 mM), malate (10 mM), lactate (5 mM), glutamate (5 mM) in U251 wt and U251 IDH1m cell lines in D5030 medium by Seahorse measurements, *:*p* < 0.05 **:*p* < 0.01. **b** Effects of 2-HG on glutamine and GABA oxidation in U251 wt, IDH1m and 2-HG-treated wt cells (the oxidation was measured after with or without 2-HG (4 mM) pre-treatment (72 h) in D5030 by Seahorse measurements) *:*p* < 0.05, **:*p* < 0.01. **c** GABA metabolism related enzyme (GAT1 - GABA transporter 1; SSADH - succinic semialdehyde dehydrogenase) expressions; glutamine metabolism related enzyme expressions (glutaminase, ASCT2 - alanine, serine, cysteine-preferring transporter 2); and acetate consumption related ACSS2 (acetyl-CoA synthetase 2) enzyme expression in U251 wt, IDH1m and 2-HG (4 mM) treated cells (72 h) using Western blot analyses. **d** Different growing characteristics of U251 wt and U251 IDH1m cells using long-term (3-week-long) 2-HG (4 mM), GABA (5 mM) and combination treatments in DMEM high glucose. After 3-week long-term continuous treatment (intermittently passaging 2 or 3 times per week) 2.5 × 10^3^ cells/100 μl were plated for 72 h and terminated by SRB tests. Data show the average of relative proliferations (untreated U251 wt – 100%) *: *p* < 0.05

Beside glucose and glutamine, other substrates were also involved to study different substrate oxidations by Seahorse technique using wt, IDH1m and 2-HG-treated U251 wt cells. No differences between U251 wt and U251 IDH1m cells were detected with respect to lactate, citrate or acetate oxidations. In contrast, decreased oxidation of glutamine, glutamate and malate was measured in IDH1m cells (Fig. 4a). 2-HG-treated U251 wt cells mimicked IDH1m cells regarding oxidation (Fig. 4b). Enzyme expressions related to glutamine and acetate consumption were detected by Western blot (Fig. 4c).

Intriguingly, significant GABA oxidation was detected in U251 wt cells (OCR was elevated with 20%) (Fig. 4a). However, IDH1m cells did not oxidise GABA and this could be mimicked in 2-HG treated U251 wt cells (Fig. 4b). The pro- or anti-proliferative effects of short- and long-term GABA, 2-HG and GABA+ 2-HG combination treatments were also tested in U251 wt and IDH1m cells. Long-term GABA treatment (for more than 3 weeks) significantly enhanced the proliferation of U251 wt cells, whereas significantly lower increase in proliferation was found in IDH1m cells (Fig. 4d). While 2-HG could not significantly reduce the proliferation of the long-term GABA treated IDH1m cells, 2-HG combination treatments reversed long-term GABA effects in the wild-type cells (Fig. 4d). Short-term (72 h) GABA, 2-HG treatments and their combination showed similar tendency in wild-type cells, however, no significant effect could be observed (data not shown).

**Fig. 5:**
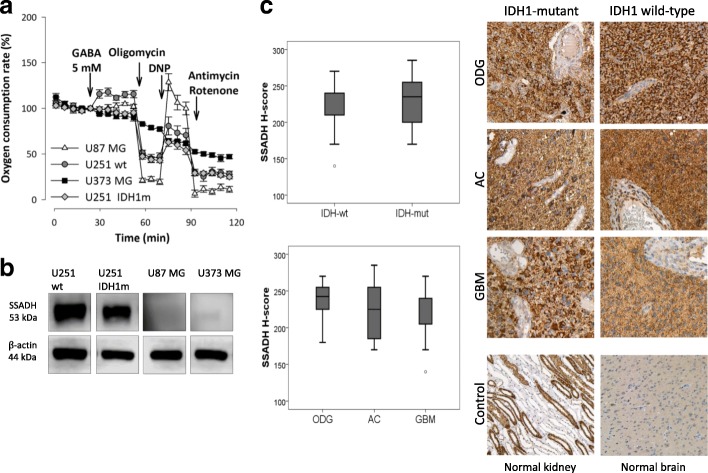
GABA oxidation and SSADH expression differences in glioma cell lines; and common SSADH expression in human glioblastoma/glioma samples. **a** Oxygen consumption rates after using GABA - as an energy substrate - in U251 wt, U251 IDH1m, U87 MG and U373 MG cells in D5030 (representative oxygen consumption curves - Seahorse measurements). **b** SSADH expression in U251 wt, U251 IDH1m, U87 MG and U373 MG cells (representative Western blot results)*.* **c** Summarised data about the evaluation of SSADH expressions in wt and IDH1m human samples (upper diagram) and in different human glioma types (lower diagram) and representative IHC stainings with HE counterstaining (DAB-brown staining, magnification 200×; normal human brain tissue and kidney were used as controls). ODG-oligodendroglioma, AC-astrocytoma and GBM-primer glioblastoma

According to the Human Protein Atlas (http://www.proteinatlas.org) database, U251 cells do not express GABA receptor subunit mRNAs (GABRA 1–6, GABRB1–3, GABRR1–3, GABRE and GABRQ), excluding receptor mediated effects of GABA. However, U251 cells do express SSADH and GAT1 proteins as shown by Western blot. Of note, SSADH and GAT1 protein level differences in IDH1m and 2-HG-treated U251 wt cells were also found (Fig. 4c.). Noteworthy, U87 MG and U373 MG did not express SSADH. Accordingly, these cells did not oxidise GABA (Fig. 5a and b). To test the potential role of GABA shunt and SSADH activity in the survival and the proliferation of SSADH expressing U251 wt cells, we tested the effect of vigabatrin (GABA transaminase inhibitor). Vigabatrin reduced the proliferation of glioma cells, as we could see this slight decrease in U251 wt cell cultures, as well. In U251 wt cells, vigabatrin could lower GABA oxidation, and in case of the presence of vigabatrin, GABA addition was not able to influence the cellular proliferation positively (Additional file 3: Figure S3). The exact role of SSADH in the detected GABA-dependent pro-proliferative effect needs examinations using specific SSADH inhibition in further studies.

### SSADH overexpression in human glioma cases

In vitro cell lines do not necessarily reflect clinical tumours in all aspects [37]. Therefore, SSADH expression was studied in human glioma biopsies and peritumoural normal brain tissues (both cerebrum and cerebellum) by immunohistochemistry. In peritumoural cerebral tissues, high expression with H-score > 150 was observed in the cortical region, however, white matter astrocytes showed low to moderate expression (150 > H-score). In the cerebellum, moderate to high expression was observed in both the molecular and the granular cell layers similarly to the Purkinje cells; we recorded that cells have low SSADH staining intensity (0/+) at non-tumoural areas. We could detect a moderate expression level in astrocytes of white matter in situ and 3+ positivity in glioma cells. Based on our results, SSADH protein level is higher in glioma cells than in normal tissues in almost all studied cases; therefore, we could not find any differences between IDH-mutant and wild-type cases at this level. In the glioma biopsies, high SSADH expression was characteristic for all histological subtypes (Fig. 5c). High SSADH expression was found in 97% of cases. This SSADH expression showed no associations with clinicopathological parameters such as age, gender, tumour type, grade or IDH mutation status.

## Discussion

Our main aim was to characterise the bioenergetic differences related to IDH mutations by using an isogenic cell line pair with and without the IDH1 R132H mutation.

The effects of epigenetic and other (e.g. metabolic) alterations caused by IDH1 mutation have been described previously [38–40]. However, few data are available about the bioenergetic consequences of IDH1 mutation and their role in growth and survival of tumour cells [25, 41].

Based on our Seahorse measurements (oxygen consumption and extracellular acidification), higher oxygen consumption is strongly correlated to mutant IDH1-produced 2-HG. In addition, after using 2-HG treatment in wild-type cell cultures, the respiration was increased to similar level as in IDH1-mutant cells. Alterations of intra- and extracellular 2-HG besides Krebs cycle metabolite levels were observed. These observations suggest the presence of metabolic compensatory mechanisms in these cells. Our results confirmed, that the Warburg phenotype is dominant in IDH1 wild-type cells, whereas IDH1-mutant cells prefer oxidative phosphorylation using substrates other than glucose. Khurshed et al. published that glycolytic enzyme mRNA expression levels are higher in IDH wild-type tumour tissues and the expression levels of TCA related enzymes are higher in IDH1-mutant human cases [20]. It was also described that mutant IDH1 enzyme was correlated with high mitochondrial density and increased mitochondrial activity in oligodendroglioma cell line xenografts in vivo [41].

In our study, the significantly reduced glutamate level in IDH1-mutant cells could correlate to enhanced glutaminolytic pathway (glutamine-glutamate-aKG-2-HG axis). This was not observed in U251 wt cells after 2-HG incubation, suggesting that glutamatolysis in IDH1-mutant cells is related to the necessity to replenish aKG levels, rather than by 2-HG induced effects [16]. Previous spectrometric analyses have shown and highlighted that the most important source of 2-HG is glutamine, and only low labelling could derive from glucose due to limited glucose turnover [26]. Glutamine was the main source of 2-HG in our experiments using ^13^C-labelled substrates in 1-h-and 24-h-labelling periods which is in line with previous report [23]. Glucose and acetate also contributed to 2-HG-labelling in 24-h ^13^C-labelling experiments. This potential contribution of acetate substrate in significant 2-HG ^13^C-labelling after 24 h was detected first in our experiments. Previous TCGA-based analyses suggest that glucose and acetate are preferential substrates in IDH wild-type, rather than IDH-mutant gliomas [20]; therefore, the relative contribution of glucose and acetate in clinical IDH-mutant gliomas needs further investigations.

It has been shown in in silico studies that IDH1-mutant gliomas have significantly higher lactate dehydrogenase B expression comparing to wild-type tumours; and higher lactate oxidation was hypothesised in IDH1 mutation bearing cells [20]. In our observations, we could not detect significant differences in lactate and acetate oxidation between wild-type and IDH1-mutant glioma cells. However, our results show that glutamine, glutamate and GABA energy substrates could significantly increase oxygen consumption rate in IDH1 wild-type U251 glioma cells (approximately 20% increase in OCR), but not in their IDH1-mutant counterpart cells. These suggest that GABA and glutamine can have an important role in energy production through substrate oxidation preferentially in IDH1 wild-type gliomas.

Dependency on glutamine and/or glutamate of IDH-mutant gliomas has been reported previously [21, 23]. Our current study has demonstrated for the first time, that GABA oxidation (using GABA as an energy substrate) via the activity of SSADH can produce energy, explaining our finding the GABA stimulated proliferation of U251 wild-type cells. However, emerging data about the exact mechanism of GABA receptor mediated pro-proliferative effects are still unclear and contradictory [42, 43]. The detected pro-proliferative effect was not the result of GABA-receptor stimulation [44] as U251 cells lack expression of these. Based on our results, 2-HG could reverse the effect of GABA on proliferation. In line with the absence of SSADH expression in U87 MG and U373 MG, these cell lines were not able to oxidise GABA. Recent publications highlight the importance of GABA shunt in primer and metastatic brain tumours. The heterogeneity in cell lines with respect to SSADH expression and GABA oxidation warrants further investigation towards GABA use in human glioma cases and in other tumours which may rely on the effect of GABA use [28, 29, 45, 46]. Our IHC studies reveal that both IDH-mutant and wild-type gliomas express high levels of SSADH in contrast to normal brain, suggesting certain role for GABA in growth and survival in clinical tumours. This correlates to data of Human Protein Atlas, where 90% of studied human gliomas showed moderate or high SSADH expression which indicates a significantly higher expression than normal brain tissue.

Related to these El-Habr and his co-workers have demonstrated that the accumulation of gamma-hydroxybutyrate (by-product of GABA in central nervous system) and the related SSADH downregulation contribute to a less aggressive phenotype in glioblastoma cases. They found higher SSADH protein expression in glioma cells than in normal brain tissue. They also described that downregulation, lowered expression and inhibited function of SSADH (shRNA silencing and gamma-hydroxybutyrate – structurally shows similarities to aKG and 2-HG – treatment) correlated to lower proliferation capacity of glioma cells in vitro and in vivo [47]. Regarding to high-grade gliomas which showed lower SSADH expression at mRNA level in TCGA database, in our study (47 cases were analysed), we could not confirm this difference at protein level. Based on our and others’ results, SSADH protein level is higher in almost all glioma cells than in normal tissues. These suggest that SSADH protein overexpression at tissue level could have special tumour cell survival and growth promoting effect in both IDH-mutant and wild-type cases. To clarify the role of GABA metabolism, SSADH expression and functions need further studies using both mRNA and protein expression studies with patient follow-up. In these studies, overall survival data evaluations and if it is possible some SSADH function related analyses should be performed.

Our findings suggest that 2-HG may contribute to a less aggressive phenotype through its inhibitory effect on GABA oxidation. The detected SSADH overexpression in human cases could support GABA metabolism through either GABA oxidation or alternative GABA consumptions. Regarding to our and others’ results, GABA metabolism of gliomas might be a possible novel entry point for therapy, especially in glioma patients with poor prognosis and limited treating opportunities.

## Conclusions

As discussed above, evaluating the dominant bioenergetic pathways, preferred substrates (such as glutamine or GABA) which can support tumour growth could help to understand metabolic reprogramming and adaptation facility better in glioma cells, and to seek new alternatives for targeted therapy in this incurable aggressive disease, respectively.

## Additional files

Additional file 1: **Figure S1.** Simplified scheme of the examined bioenergetic pathways in the studied IDH1 wild-type and mutant glioma cells. In our study, different energy substrate oxidation and metabolic enzymes relating to the following bioenergetic pathways were analysed regarding the role of IDH1 mutation. Energy substrate oxidation: glucose, glutamine, citrate, GABA, acetate, malate, lactate and glutamine were measured by Seahorse technique. Energy metabolites - succinate, fumarate, malate, citrate, α-ketoglutarate, glutamate and 2-hydroxyglutarate were determined by liquid chromatography-mass spectrometry. Several protein expressions were measured by Western blot analysis or immunohistochemistry - Glycolysis: hexokinase 2 (HK2), phosphofructokinase P (PFKP), Glutaminolysis: alanine, serine, cysteine-preferring transporter 2 (ASCT2), glutaminase (Gls), GABA shunt: GABA transporter (GAT1), succinic semialdehyde dehydrogenase (SSADH), acetate consumption: acetyl-CoA synthetase 2 (ACSS2). Other abbreviations can be found in the figure: GLUT1: glucose transporter 1, IDH: isocitrate dehydrogenase, LDH: lactate dehydrogenase, MCT1: monocarboxylate transporter 1, OAC: oxaloacetate, SSA: succinic semialdehyde. (PDF 348 kb)

Additional file 2: **Figure S2.** Extracellular 2-HG levels after ^13^C-substrate labellings detected by LC-MS in U251 IDH1m cells. a., 2-HG pool after 24 h following ^13^C-substrates incubation: 4 mM U-^13^C-glutamine labelled intra- and extracellular 2-HG. b., 10 mM U-^13^C-glucose labelled extracellular 2-HG in D5030. c., 10 mM 2-^13^C-acetate labelled 2-HG in D5030. Unlabelled 2-HG did not contain incorporated ^13^C atoms, M + 1/2/3/4/5 = mass number increased with 1/2/3/4 or 5 ^13^C atoms in 2-HG from different labellings (the low rate of M + 4 is not visible in the figure). The labelling conditions were given in the legends of Fig 3. (PDF 197 kb)

Additional file 3: **Figure S3.** Vigabatrin abolished the pro-proliferative effect of GABA a., The effect of GABA (5 mM), vigabatrin (0.6 mM) and GABA+vigabatrin on the proliferation of U251 wt glioma cells. SRB and Alamar Blue (AB) proliferation assays were used in 24-h treated cell cultures; b., Alterations in cell numbers (U251 wt cells) followed in every 4-day passage using 3-week continuous treatment, the average cell numbers were calculated from triplicates. (PDF 198 kb)

## Abbreviations

2-HG: D-2-hydroxyglutarate
ACSS2: Acetyl-CoA synthetase 2
AML: Acute myeloid leukaemia
ASCT2: Alanine, serine, cysteine-preferring transporter 2
ATPB: β-F1-ATPase
FH: Fumarate hydratase
GABA: Gamma-aminobutyric acid
GAT1: GABA transporter 1
Gls: Glutaminase
HK2: Hexokinase 2
ICC: Intrahepatic cholangiocarcinoma
IDH: Isocitrate dehydrogenase
IDH1m: IDH1-mutant
PFKP: Phosphofructokinase P
SDH: Succinate dehydrogenase
SSADH: Succinic semialdehyde dehydrogenase
TCA: Tricarboxylic acid
