# Supplementary figures and images for: GABA, glutamine, glutamate oxidation and succinic semialdehyde dehydrogenase expression in human gliomas
Source: J Exp Clin Cancer Res. 2018 Nov 7;37:271. doi: 10.1186/s13046-018-0946-5 (PMC6223071; doi:10.1186/s13046-018-0946-5)

Figure S1

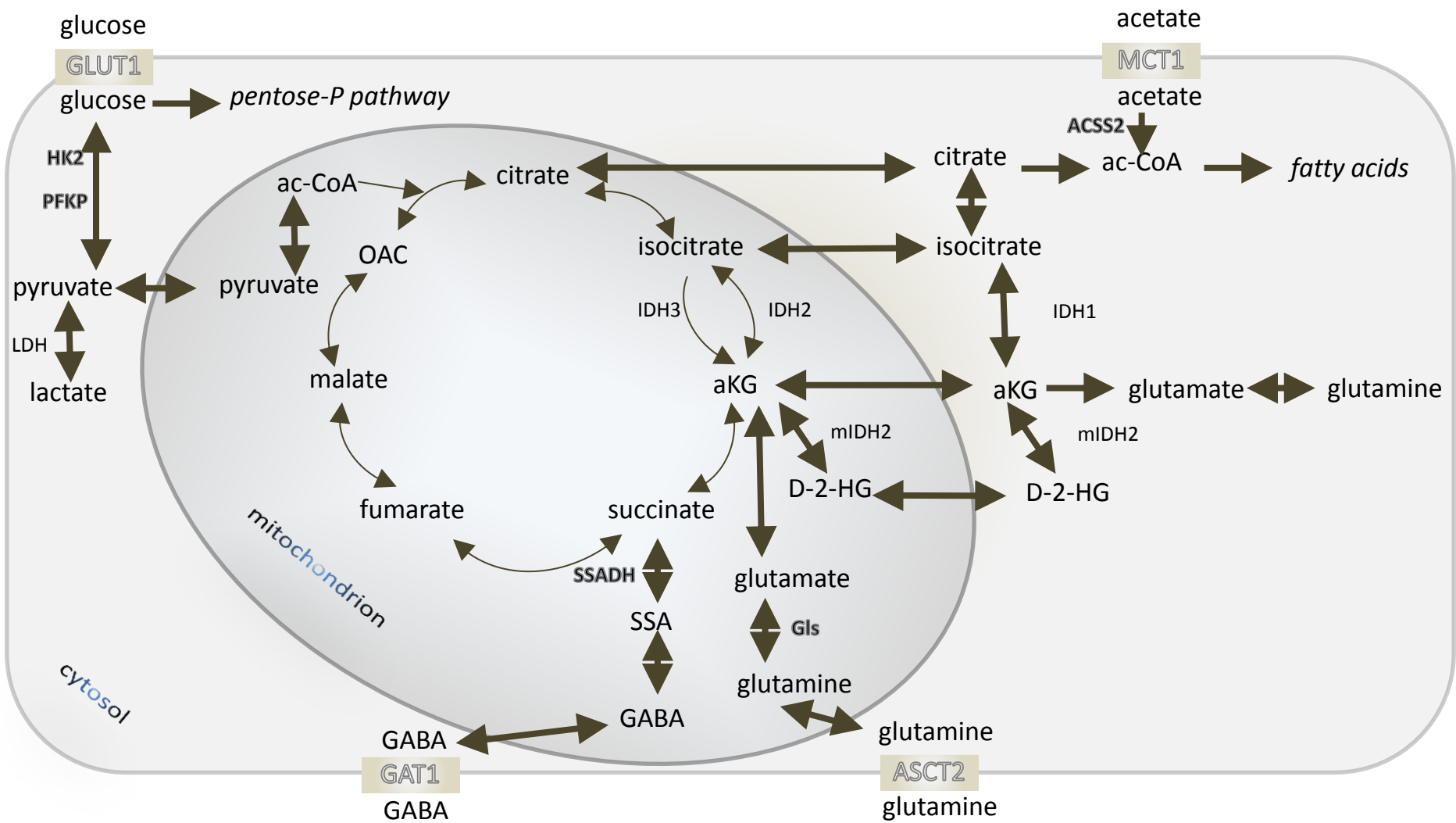

Supplement: Supplementary file 1 — Figure S1. Simplified scheme of the examined bioenergetic pathways in the studied IDH1 wild-type and mutant glioma cells. In our study, different energy substrate oxidation and metabolic enzymes relating to the following bioenergetic pathways were analysed regarding the role of IDH1 mutation. Energy substrate oxidation: glucose, glutamine, citrate, GABA, acetate, malate, lactate and glutamine were measured by Seahorse technique. Energy metabolites - succinate, fumarate, malate, citrate, α-ketoglutarate, glutamate and 2-hydroxyglutarate were determined by liquid chromatography-mass spectrometry. Several protein expressions were measured by Western blot analysis or immunohistochemistry - Glycolysis: hexokinase 2 (HK2), phosphofructokinase P (PFKP), Glutaminolysis: alanine, serine, cysteine-preferring transporter 2 (ASCT2), glutaminase (Gls), GABA shunt: GABA transporter (GAT1), succinic semialdehyde dehydrogenase (SSADH), acetate consumption: acetyl-CoA synthetase 2 (ACSS2). Other abbreviations can be found in the figure: GLUT1: glucose transporter 1, IDH: isocitrate dehydrogenase, LDH: lactate dehydrogenase, MCT1: monocarboxylate transporter 1, OAC: oxaloacetate, SSA: succinic semialdehyde. (PDF 348 kb) [file 13046_2018_946_MOESM1_ESM.pdf]

Figure S2

2-HG labelling after 24-h  $^{13}\text{C}$ -substrate incubation (U251 MG IDH1m cells)

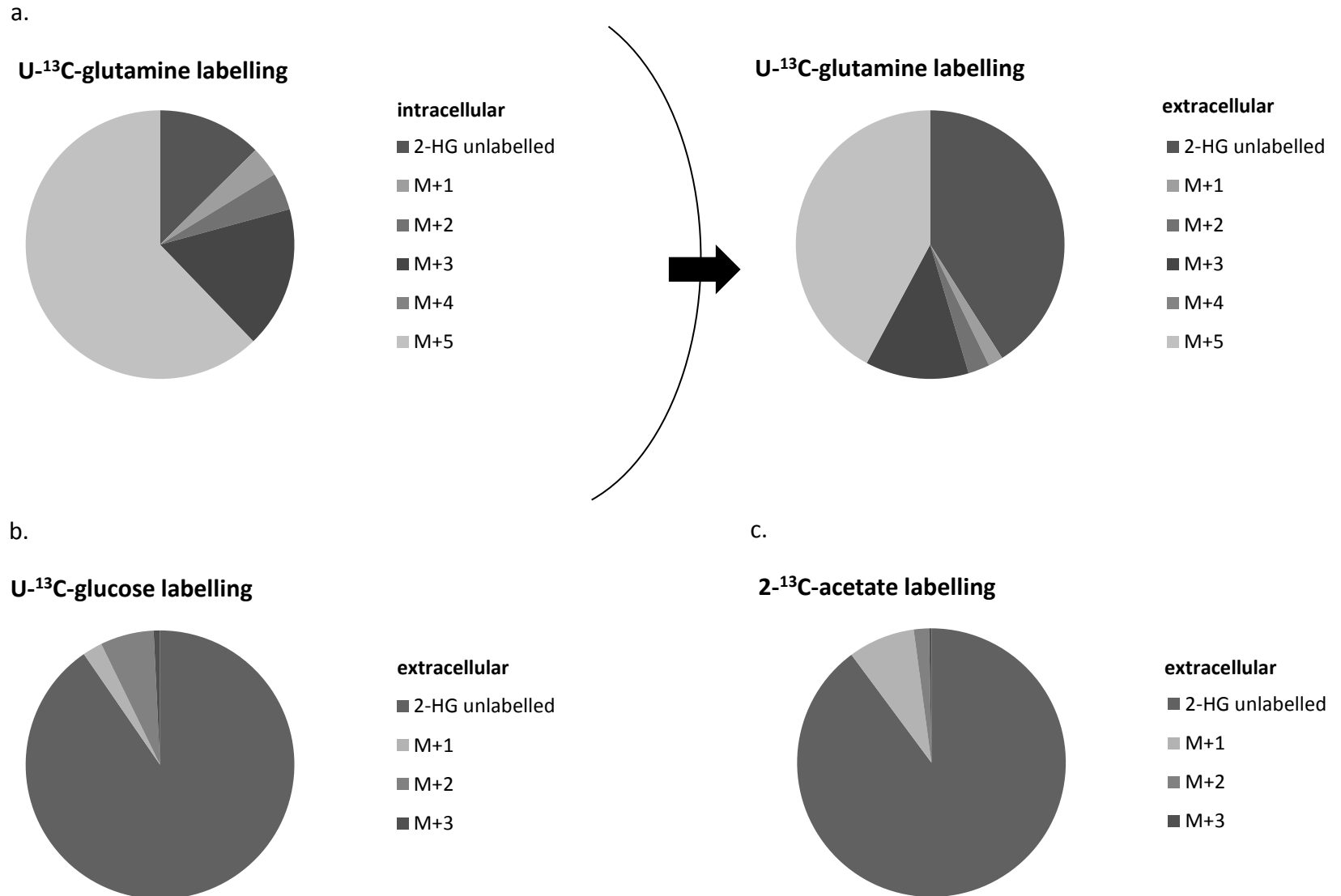

Supplement: Supplementary file 2 — Figure S2. Extracellular 2-HG levels after 13C-substrate labellings detected by LC-MS in U251 IDH1m cells. a., 2-HG pool after 24 h following 13C-substrates incubation: 4 mM U-13C-glutamine labelled intra- and extracellular 2-HG. b., 10 mM U-13C-glucose labelled extracellular 2-HG in D5030. c., 10 mM 2-13C-acetate labelled 2-HG in D5030. Unlabelled 2-HG did not contain incorporated 13C atoms, M + 1/2/3/4/5 = mass number increased with 1/2/3/4 or 5 13C atoms in 2-HG from different labellings (the low rate of M + 4 is not visible in the figure). The labelling conditions were given in the legends of Fig 3. (PDF 197 kb) [file 13046_2018_946_MOESM2_ESM.pdf]

Figure S3

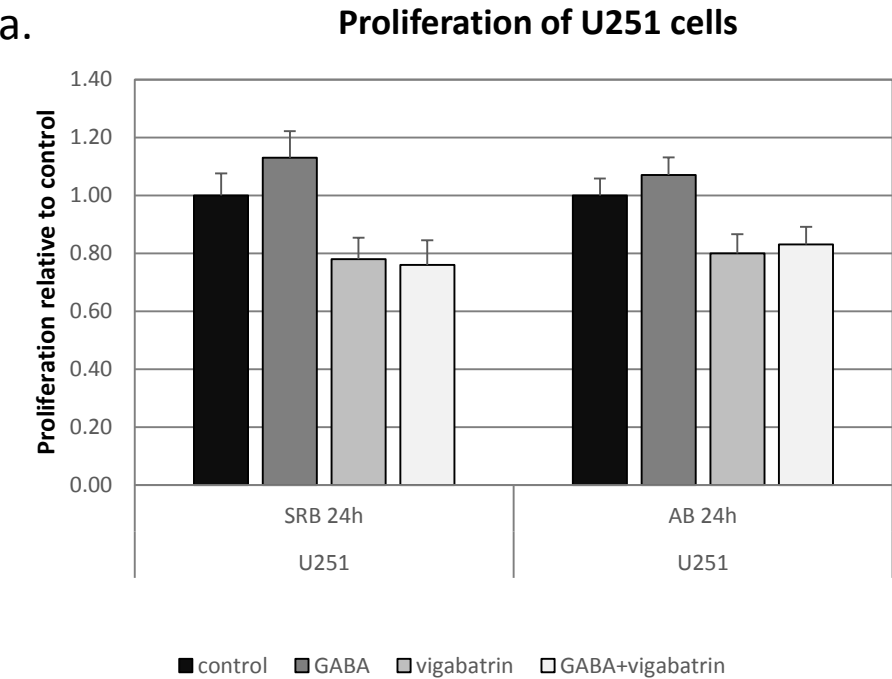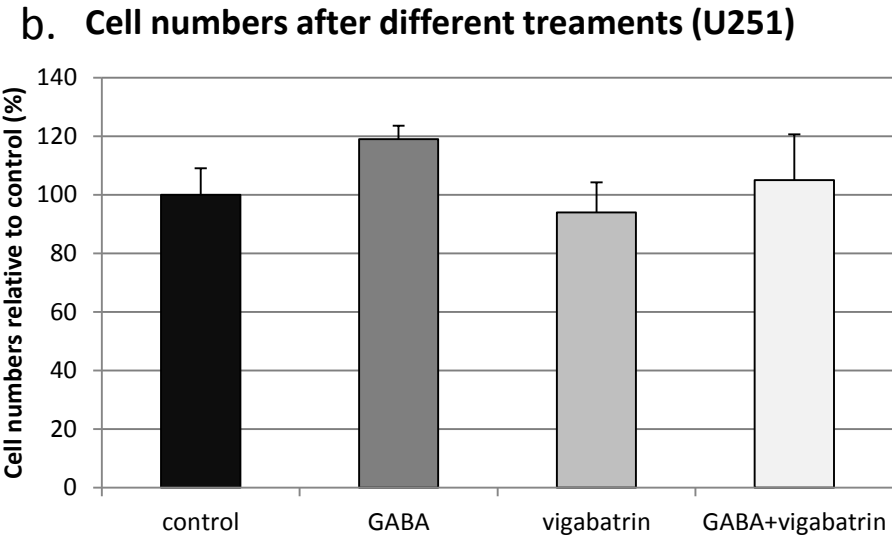

Supplement: Supplementary file 3 — Figure S3. Vigabatrin abolished the pro-proliferative effect of GABA a., The effect of GABA (5 mM), vigabatrin (0.6 mM) and GABA+vigabatrin on the proliferation of U251 wt glioma cells. SRB and Alamar Blue (AB) proliferation assays were used in 24-h treated cell cultures; b., Alterations in cell numbers (U251 wt cells) followed in every 4-day passage using 3-week continuous treatment, the average cell numbers were calculated from triplicates. (PDF 198 kb) [file 13046_2018_946_MOESM3_ESM.pdf]
